# The Effect of Perceived Overqualification on Creative Performance: Person-Organization Fit Perspective

**DOI:** 10.3389/fpsyg.2021.582367

**Published:** 2021-05-13

**Authors:** Man Zhang, Fan Wang, Na Li

**Affiliations:** ^1^Party School of Anhui Provincial Committee of C.P.C., Hefei, China; ^2^School of Management, Shanghai University, Shanghai, China

**Keywords:** perceived overqualification, creative performance, employee development-oriented organizational culture, organizational identification, P-O fit

## Abstract

In today’s business world, the phenomenon of overqualification is widespread. Organizations need to consider – how to motivate the overqualified employees to utilize their qualifications, for example, promoting creative performance. Based on person-organization (P-O) fit theory, this study explored when and how employees, who feel overqualified can engage in creative performance. Data were collected from 170 supervisor-subordinate dyads of 41 groups in 10 manufacturing companies across two timepoints in China. Results revealed that (1) perceived overqualification is positively related to organizational identification when employee development-oriented organizational culture is strong but negatively related to organizational identification when employee development-oriented organizational culture is weak, (2) organizational identification is positively related to creative performance, (3) the indirect relationship between perceived overqualification and creative performance via organizational identification is moderated by employee development-oriented organizational culture. We provide several theoretical contributions to the overqualification literature and make some suggestions to motivate the overqualified employees to use their surplus qualifications within the organizations.

## Introduction

In global organizations, a large number of employees report that their skills, experiences, or education exceed their work requirements (Feldman, 1996; Quintini, 2011; Hu et al., 2015; Erdogan et al., 2018b). The phenomenon that employees feel overqualified seems to be increasingly prevalent around the world. It is estimated that 20–25% of the employees in the United States feel overqualified (Feldman and Turnley, 1995), while the proportion in Britain and Canada is higher (Battu and Sloane, 2002). Some scholars believe the proportion of overqualified employees is likely to increase in the future and call for attention to the impact of perceived overqualification on performance (Liu and Wang, 2012). The literature to date about the results of perceived overqualification has obtained mixed findings. For instance, some scholars pointed out that perceived overqualification related to poor performance, low organizational commitment, low job satisfaction, and high turnover intentions (Kristof, 1996; Bolino and Feldman, 2000; Erdogan and Bauer, 2009; Maynard and Parfyonova, 2013). However, others alleged that overqualified employees might perform better than just-qualified employees because they possess higher levels of workability and work experience (Holtom et al., 2002; Fine, 2007; Erdogan and Bauer, 2009). The laters’ argued that perceived overqualification positively related to performance. Given this mixed evidence, most literature suggested that the inconsistent results of perceived overqualification likely depend on contextual factors (Erdogan et al., 2018a). Therefore, it is essential to explore how organizations can encourage overqualified employees to use their surplus qualifications constructively (i.e., engage in creative performance). Creative performance, as one of the most important factors affecting the sustainable development of the company (Mone et al., 1998). Organizations constantly seek ways to improve creative performance and maintain a sustained competitive advantage in today’s marketplace (Carmeli et al., 2013). Moreover, the improvement of creative performance requires employees to have extra ability and skills to engage in creative activities besides their core duties. That is, overqualified employees are more likely to produce creative performance. However, the relationship between perceived overqualification and creative performance has been largely ignored. Thus, we attempted to explore the internal mechanism and contextual conditions of the perceived overqualification–creative performance link.

Perceived overqualification can be considered as a type of person-job (P-J) misfit (Van Der Vegt and Bunderson, 2005; Liu and Wang, 2012), which may harm employees’ work attitude and behavior (i.e., low creative performance) (Erdogan et al., 2018a). However, Kristof-Brown pointed out that P-J fit will interact with person-organization (P-O) fit to affect employees’ performance (Kristof-Brown et al., 2005). Hence, we assume P-O fit may improve overqualified employees’ creative performance even if the P-J misfit still exists. Employee development-oriented organizational culture encourages employees to develop their skills and provides them with opportunities for training and development, which is consistent with the personal values of overqualified employees (Tsui et al., 2006). As an effect way of P-O fit, employee development-oriented organizational culture have similarities in value consistency with organizational identification (Ashforth and Mael, 1989; Kristof-Brown et al., 2005). Therefore, we can infer that employee development-oriented organizational culture may have a great impact on the relationship between perceived overqualification and organizational identification. Further, creative performance is the product of the combination of employee desire, employee capability, and related conditions (He and Brown, 2013; Man et al., 2020; Xie et al., 2020). Overqualified employees have more abilities and experience than others to participate in creative activities. Therefore, it is critical to study the internal mechanism of employees’ creative motivation. P-O fit is an imperative influencing factor of organizational identification in the sense that people with high organizational identification may have more personal motivation in contributing to the organization (De Cremer and Van Vugt, 1999; Van Knippenberg, 2000). Hence, we assume organizational identification is the key to the internal mechanism of perceived overqualification influencing creative performance.

Taken together, grounded in the P-O fit theory, we developed a moderated mediation model and predicted the relationship between perceived overqualification and creative performance. We assumed that perceived overqualification has an indirect influence on creative performance via organization identification when employee development-oriented organizational culture is strong or weak.

This study made contributions to the present literature in the following specific ways. Firstly, we examine a key boundary condition of the relationship between perceived overqualification and creative performance. By examining employee development-oriented organizational culture as a moderator, we contribute to the overqualification literature and explicate how organizations can benefit from overqualified employees. Secondly, we contribute to P-O fit literature by testing the interaction between perceived overqualification and organizational culture on organizational identification. We found P-O fit and P-J fit have complex interactions, which may affect employees’ emotional perception and behavior. Thirdly, we try to extend the literature by explicitly examining organizational identification as a mediating mechanism in the perceived overqualification–creative performance relationship.

## Theoretical Background

Overqualification refers to the extent to which employees possess more education, experiences, or skills relative to the requirements of their job (Maynard et al., 2006; Erdogan and Bauer, 2009; Ma et al., 2020a,b). Previous research has distinguished between perceived overqualification and objective overqualification. Objective overqualification is often judged by someone else, while perceived overqualification refers to the extent to which employees think they are overqualified (Maltarich et al., 2011). Maynard et al. (2006) argue that perceived overqualification is more suitable than objective overqualification for psychological research since perceived overqualification is considered as a proximal indicator of work attitude and behaviors (Erdogan et al., 2011; Liu and Wang, 2012). Consistent with previous researchers (Maynard et al., 2006; Erdogan and Bauer, 2009), we employ a subjective measurement of overqualification in this study. Perceived overqualification is generally regarded as an obstacle to both employment and retention. Extant studies have shown that perceived overqualification is associated with negative consequences, such as lower subjective well-being (Erdogan et al., 2018b), lower organizational commitment (Harari et al., 2017), higher perceived job insecurity (Peiro et al., 2012), and higher turnover intention (Maynard and Parfyonova, 2013). In recent years, a mounting evidence suggests that overqualified employees may make contributions to their organizations under appropriate conditions in the form of proactive behaviors (Zhang et al., 2016), performance (Deng et al., 2018) and creativity (Luksyte and Spitzmueller, 2016). Indeed, overqualified employees have more energy and time to participate in extra activities because they can accomplish their in-role tasks quickly (Liu and Wang, 2012).

In the person-environment (P-E) fit literature, overqualification is viewed as a type of person-job misfit (Maynard et al., 2006). P-E fit theory also realizes that multiple types of fit exist simultaneously, among which person-organization fit (P-O fit) is one of the most commonly studied types of fit (Kristof-Brown et al., 2005). Researchers proved that different types of fit may generate interactive effects that high fit in one area could compensate the low fit in others (Kristof-Brown et al., 2002), because individuals cognitive dissonance caused by having fit on one type would be diluted when they fit on another type (Jansen and Kristof-Brown, 2006). Based on P-O fit theory, we proposed that overqualified employees will produce creative performance through their sense of organizational identification. This is because, under the situation of employee development-oriented organizational culture, P-O fit can make up for the deficiency of P-J misfit. P-O fit indicates the congruence between employees’ identity and organizational values (Kristof, 1996), which is composed of two types: supplementary fit and complementary fit. Supplementary fit means a person and an organization possess similar or matching characteristics, while complimentary fit means that an employee has qualifications that an organization requires, and an organization offers the rewards that an individual needs. Previous literature has shown that employees who fit well with organizations are more likely to have positive work attitudes and behaviors, such as high job satisfaction, low turnover intention, high organizational identification, and more citizenship behavior (Chatman et al., 1998; Kristof-Brown et al., 2005; Farzaneh et al., 2014). Consistent with P-O fit theory and previous researches, we argue that improving the level of people-organization fit, workers who view themselves as overqualified – can enhance the employee’s organizational identification and further produce creative performance.

### The Moderating Role of Employee Development-Oriented Organizational Culture

Organizations require employees to contribute in terms of their time, effort, commitment, and qualifications. At the same time, the organization provides employees with financial, physical, and psychological resources, as well as job-related growth opportunities (Kristof-Brown et al., 2005). According to the two aspects of P-O fit, complementary fit emphasizes to meets the demands of both sides, while supplementary fit refers to the characteristic congruence between employees and organization (Kristof, 1996). Therefore, the organization and employees can achieve a high level of fit in a certain situation (e.g., employee development-oriented organizational culture). Previous literature regards perceived overqualification as a P-J misfit, which leads to negative outcomes (Kristof-Brown et al., 2005; Liu and Wang, 2012). However, extant literature has shown that P-O fit can interact with P-J fit to affect employee cognition and behavior (Erdogan et al., 2018a). For instance, Bills pointed out that overqualified employees will be satisfied with their situation when they fit well with the organization (Bills, 1992). Thus, we followed the P-O fit theory and proposed that employee development-oriented organizational culture is a key influential factor of the relationship between perceived overqualification and employees’ organizational identification.

When employee development-oriented organizational culture is strong, overqualified employees may fit well with the organization even if the P-J misfit still exists. From the supplementary fit dimension, overqualified employees attach importance to their qualifications, and opportunities for extensive training/development that organizations respect for employees’ qualifications (Bailey et al., 2001). From the complementary fit dimension, comprehensive opportunities are important resources that are demanded by employees (Kristof, 1996). The organization provides opportunities to convey the information that the organization regards employee development as an important catalyst (Wayne et al., 2002). Scholars have pointed out that employees have the qualifications which are of value by organizations, while organizations can provide employees with what they want (i.e., development opportunities and work resources) (Cable and Edwards, 2004). Therefore, when employee development-oriented organizational culture is strong, overqualified employees may fit well with the organization.

Employees who fit well with the organization are likely to have more positive work attitudes and behaviors (Verquer et al., 2003). Besides, previous studies have shown that employees possess a stronger sense of organizational identification when the extent of P-O fit is greater (Edwards and Rothbard, 2000; Verquer et al., 2003; Hoffman and Woehr, 2006; Amos and Weathington, 2008). Hence, employees who feel overqualified may contribute to such P-J misfit situation for understandable reasons such as economic environment instead of organizational reasons. Thus, we infer that perceived overqualification is positively related to organizational identification when employee development-oriented organizational culture is strong.

In contrast, when employee development-oriented organizational culture is weak, overqualified employees might feel that their qualifications are wasted and a strong misfit with the organization. From the supplementary misfit dimension, overqualified employees pay more attention to their development opportunities and make full use of the qualifications. However, the culture of the organization does not attach importance to the growth of employees. From the complementary misfit dimension, employees provide organizations with job-related skills, education, and other qualifications, while organizations do not provide employees with jobs that meet their expectations. Taken together, when employee development-oriented organizational culture is weak, overqualified employees may misfit with the organization and lead to low organizational identification.

H1: The relationship between perceived overqualification and organizational identification is moderated by employee development-oriented organizational culture such that this relationship is positive when employee development-oriented organizational culture is strong but negative when it is weak.

### Organizational Identification and Creative Performance

Employees who have high levels of organizational identification may be internalized by the organization, and they are likely to do something beneficial to the development of the organization (Dutton et al., 1994; De Cremer and Van Vugt, 1999). Ashforth and Mael (1989) pointed out when the employees consider the organization to be their own, they will increase their cognitive, emotional, and behavioral investment in the organization. Hence, a high level of organizational identification is correlated with some important workplace outcomes (Van Dick et al., 2004; Cole and Bruch, 2006). Thus, we assume people with high levels of organizational identification may actively participate in creative activity.

Previous literature has shown that organizational identification may positively contribute to employees’ performance (Walumbwa et al., 2008; Weiseke et al., 2008). Moreover, scholars pointed out that when employees have a strong sense of organizational identification, they are likely to improve their work and generate new ideas (Lipponen et al., 2008). Similarly, it is believed that organizational identification can not only promote employees to engage in creative activities but also stimulate the internal motivation to create new things (Bailey et al., 2001; Hirst et al., 2009). Thus, we infer that the relationship between organizational identification and creative performance is positive.

H2: Organizational identification is positively related to creative performance.

### A Moderated Mediational Model

As we mentioned before, creative performance is the product of the combination of three factors: Employee desire, employee capability, and related conditions (He and Brown, 2013). Overqualified employees have unused resources beyond their normal work, which can be used for creative activities. Besides, overqualified employees spend less time completing their work than their colleagues because of their superior education, skills, and experience, giving them more time to participate in creative activities (Liu and Wang, 2012). Thus, we find that for overqualified employees, the key to improving their creative performance is motivating their desire to create. Through the analysis of the front part of the paper, we found perceived overqualification may leading to contrary organizational identification on a different level of employee development-oriented organizational culture, and organizational identification, which may be positively related to creative performance.

In sum, the logical analysis sketches a complex framework that organizational identification may mediate the relationship between perceived overqualification and creative performance, and employee development-oriented organizational culture may moderate this indirect relationship. Specifically, when employee development-oriented organizational culture is strong, overqualified employees may fit well with the organization, and organizational identification is improved in the process. Promoted by organizational identification, overqualified employees are more willing to serve and reward the organization by utilizing surplus resources to engage in creative activities. On the contrary, if employee development-oriented organizational culture is weak, overqualified employees may have low levels of organizational identification due to P-O misfit, which may reduce their creative performance. In general, employee development-oriented organizational culture and organizational identification play an important role in influencing overqualified employees’ creative performance.

H3: The indirect relationship between perceived overqualification and creative performance via organizational identification is moderated by employee development-oriented organizational culture and this relationship is positive when employee development-oriented organizational culture is strong but negative when it is weak.

**FIGURE 1:**
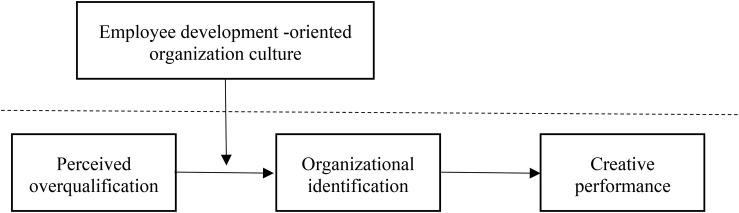
Study model.

The main study variables and hypothetical links between them are graphically shown in Figure 1.

## Materials and Methods

### Sample and Procedure

We collected data from 10 companies operating in South China, from the industries of manufacturing. To effectively determine the causal relationship, our study adopted the multi-source (employees and their immediate supervisors), multi-time (two points in time T1 and T2) method to survey data. With the assistance of the company human resources department manager, we randomly invited 50 team leaders as well as their 250 subordinates from 10 companies to participate in our study.

Employees rated their perceived overqualification and demographic variables such as age, gender, educational level in time 1 and organization identification and employee development-oriented organizational culture in time 2, their supervisors evaluated subordinates’ creative performance in time 2. The T2 surveys were distributed 2 months after T1 which helps reduce potential common method variance (Podsakoff et al., 2003). Some researchers have noted the importance of choosing an appropriate time lag because it “reduces the risk of erroneously concluding” (Meier and Spector, 2013; Eisenberger et al., 2014). Prior longitudinal studies on creative performance revealed a wide disparity in timing on data collection intervals, ranging from 1 week (e.g., Ozturk and Karatepe, 2018) to 6 months (e.g., Li et al., 2020). We chose a time lag of 2 months for several reasons. Firstly, although previous studies have shown that creativity-related outcomes can change over a few weeks (Ozturk and Karatepe, 2018). For example, Lin et al. (2017) collected data with a time lag of 1 week between each survey to examine the effects of perceived underemployment on employees’ creativity. However, unlike creativity that could result when the employees engage in task crafting every day, creative performance needs the accumulation of creative activities in a longer period (Lin et al., 2017). As our model deals with a psychological process and its effects on employee creative performance, so when employees perceived overqualification, it may have an impact on the internal psychology of employees with the gradual implementation of employee development-oriented organizational culture. And with the accumulation of creative activities, the creative performance will change in a long enough period. Besides, a 2-months study span was long enough for changes in creative performance. Although some studies employing longer lags (e.g., 6 months; Tierney and Farmer, 2011) are informative, some have suggested that shorter intervals between assessments must be considered. Li et al. (2020) suggested that the use of shorter intervals is because studies with longer lags may underestimate the true relationship between the predictor and creative performance. Furthermore, in the research, the effect of perceived overqualification on Harmonious Passion (similar psychological processes to ours), Cheng et al. (2020) suggested that the interval of 2 months is appropriate. Considering these factors, we allowed for a 2-months lag in our data collections, expecting that it would permit adequate time for individual changes in organizational identification and creative performance.

We conducted the study in two waves and combined the paper-and-pencil version and on-line version. Using name lists provided by the human resources department manager, we assigned a unique code number to each participant and ensured that each survey listed the correct number to match surveys across time. The code number is composed of the company abbreviation, the employee/leader code (E or L), and work number (e.g., ZHE119034 and ZHL118032). Supervisor questionnaires also included a code number for each employee instead of names. We also provided each supervisor with a list of names matched to code numbers to facilitate their ratings.

Due to the uncertainty of employee access to computers, to ensure the amount of data we collect, we also use paper-based surveys to gather data in time 1. We invited 250 subordinates who were divided into several groups to fill the questionnaire in the meeting rooms and then issued questionnaires through the one-to-one correspondence way between questionnaire number and employees’ work number. We also explained the purpose of the study, emphasizing that data was only used for scientific research. More importantly, we encourage participants to provide a valid e-mail address and actively participate in the second research online. At the end of the survey, we gave each participant about $5 in cash and promised that we will give $10 for the second survey in 2 months. Among the 250 questionnaires collected on-site, a total of 236 employees provided complete data (including email addresses), with a response rate of 95.40%.

To improve our research efficiency, we use an online survey website in T2. With the help of Wenjuanxing website^1^, the Chinese version of Qualtrics, each questionnaire had a unique questionnaire ID automatically generated within Wenjuanxing. Based on the collected information of the paper version of the questionnaire, we made one-to-one correspondence between the questionnaire number, work number, email address, and the Wenjuanxing ID and recorded them. We then sent the second questionnaire link to the 236 subordinates who provided a valid email address and their 50 immediate supervisors whose email was provided by the HR manager. We also asked the participants for their Alipay accounts in the email and gave them a $10 reward when the collection ended. Among those who received the link, 211 subordinates and their 41 immediate supervisors answered the online questionnaire with a response rate of 89.40% and 82%. Further, no significant difference was identified in a *t*-test between demographics for all employees were collected from both T1 and T2, and checks for non-response bias were conducted. There were no differences between invitees who did and did not respond in terms of gender (*t* = 0.81, *p* > 0.05), and perceived overqualification (*t* = 0.66, *p* > 0.05). Hence, we could guarantee that all survey listed were matched between subordinates and supervisors across time.

We excluded questionnaires with no responses on several variables of interest, T1 questionnaires with incorrect or no matching information, and T2 questionnaires from supervisors with less than three respondents. Finally, 170 subordinates’ questionnaires and 41 supervisors’ questionnaires were returned, which represented a response rate of 68 and 82%. On average, a supervisor had four employees. In the sample of subordinates, 58.8% were male, 90.6% had a bachelor’s degree or above. The average age of employees was 37 years. The employees’ average monthly income was 4006 Yuan approximately. Also, we ran our model at different cutoffs. The results did not change in direction or significance when we included data from employees with incorrect or no matching information or a team with less than three respondents (*N* = 211), but some probabilities dropped to marginal significance, likely because of the reduced sample size loss of statistical power. In sum, the results indicate that respondent attrition was mainly random.

### Measures

Surveys were administered in Chinese. To confirm the accuracy of the translation and correct any discrepancies, we employed Brislin’s translation/back-translation procedures (Brislin, 1980). Unless otherwise indicated, we used a 5-point Likert-type scale, ranging from 1 (strongly disagree) to 5 (strongly agree).

#### Perceived Overqualification

We measured perceived overqualification using the nine-item scale developed by Maynard et al. (2006) which from the perspective of education, experience and skills to judge whether one’s qualification exceed what current job requirements. Perceived overqualification was employees’ self-evaluation, the higher the score, the higher the degree of overqualification. This scale was widely used in previous studies and has been proved to have high reliability and validity (Luksyte et al., 2020; Zhang et al., 2021). Sample items included “My education level is above the education level required by my job” “Someone with less work experience than myself could do my job as well.” Cronbach’s alpha of the scale was 0.731.

#### Creative Performance

The creative performance was measured using the five-item scale developed by George and Zhou (2002) which has been proved to have high reliability and validity. We asked supervisors to rate each of their subordinates on each item. The scale measured three dimensions of individual creative performance: the generation, promotion and realization of innovative thinking at work. Sample items included “This employee comes up with new and practical ideas to improve performance at work”; “This employee searches out new technologies, processes, techniques, and/or product ideas at work.” Cronbach’s alpha of these items was 0.843.

#### Organizational Identification

Van Der Vegt and Bunderson (2005) proposed that four items related to emotion can be used to measure organizational identity in performance related research. Consistent with prior research, we measured organizational identification using the four-item scale created by Van Der Vegt and Bunderson (2005). Sample items included “I feel as if the team’s problems are my own” and “I feel emotionally attached to my team.” Cronbach’s alpha found for this scale was 0.925.

#### Employee Development-Oriented Organizational Culture

Tsui et al. (2006) put forward scales often organizational culture which covered employee development-oriented organization culture. We used Tsui, Wang, and Xin’s five-item scale to measure employee development-oriented organizational culture (Tsui et al., 2006). Sample items included “the company/department concern for my development.”; “the company/department developing my potentials.” Cronbach’s alpha was 0.873 for this scale. Based on our theoretical hypotheses and previous studies (Erdogan et al., 2006), we aggregated the construct at the team level and diagnosed whether group consensus was evidenced through interrater agreement (rwg) and intra-class correlations (ICCs). The results indicated that the median rwg was 0.806, greater than the suggested 0.70 thresholds, suggesting that employees agreed to the degree of the development-oriented organizational culture. The ICC (1) value was 0.14, suggesting that the between-group variance in the employee development-oriented organizational culture variable was significant. Overall, the results support the team-level aggregation of this scale.

Consistent with previous research (Harari et al., 2017), this research set the individual characteristics variables that may affect organizational identification as control variables, including education, income, age, times of promotions, working years with direct supervisors, and so on.

## Results

### Descriptive Statistics and Correlations

The descriptive statistics and correlations of the variables in our study are presented in Table 1. The correlations are as expected. Perceived overqualification was negatively correlated to organization identification (*r* = −0.169, *p* < 0.05). Organization identification was positively correlated to creative performance (*r* = 0.347, *p* < 0.01).

**TABLE 1:** Means, standard deviations, and correlations.

Variables	*M*	SD	1	2	3	4	5	6	7	8	9
1. Gender	1.412	0.494									
2. Age	37.100	8.525	−0.170*								
3. Education	3.371	0.760	−0.031	−0.412**							
4. Salary	4.006	1.391	0.322**	0.156*	0.295*						
5. Promotion chances	1.047	2.215	−0.240**	0.098	0.148	0.294**					
6. Self-rated EDOOC	4.368	0.589	0.037	0.057	0.095	0.000	0.164*	(0.873)			
7. Self-rated OI	4.422	0.416	0.062	0.148	0.008	0.125	0.066	0.467**	(0.925)		
8. Self-rated POQ	2.923	0.616	0.010	0.111	−0.035	−0.128	−0.083	−0.125	−0.196*	(0.731)	
9. Sup-rated CP	4.533	0.459	−0.076	0.160*	0.050	0.145	0.071	0.372**	0.347**	−0.0347	(0.843)

*p < 0.05, **p < 0.01. CP, creative performance; EDOOC, employee development-oriented organizational culture; OI, organizational identification; POQ, perceived overqualification; Income: measured in thousands of Yuan; Gender: 1 = *male*; 2 = *female*; Education: 1 = *middle school education or less*; 2 = *high school education*; 3 = *bachelor’s degree*; 4 = *postgraduate degree*; 5 = *doctor degree*.

### Confirmatory Factor Analysis (CFA)

According to Bandalos’s recommendation (Bandalos, 2002), the parameter-to-item ratio should be above almost a certain proportion (10:1), the direct use of the original title will lead to some estimation bias. According to Rogers and Schmitt’s recommendation, this study used the item factor packaging method to deal with the four variables, and the packaging strategy adopts a high-high strategy (Rogers and Schmitt, 2004). According to the packaging results (As shown in Table 2), the four-factor model is the best (χ^2^ = 115.148, df = 48, RMSEA = 0.078, TLI = 0.868, and CFI = 0.901), and the other alternative competition models are inferior to the hypothetical models proposed in this study.

**TABLE 2:** Model fit results for confirmatory factor analyses.

Model	χ^2^	Df	χ^2^/df	TLI	CFI	RMSEA	SRMR	Δχ^2^
1. Four-factor model	115.148	48	2.399	0.864	0.901	0.091	0.078	
2. Three-factor model	192.637	51	3.777	0.729	0.791	0.128	0.090	77.489
3. Two-factor model	219.089	53	4.134	0.698	0.758	0.135	0.097	103.941
4. Single-factor model	347.275	54	6.431	0.470	0.567	0.179	0.113	232.127

*Three-factor model combined organizational identification and employee development-oriented organizational culture together. Two-factor model combined perceived overqualification, organizational identification, and employee development-oriented organizational culture together. Single-factor model combined all items together.*

### Null Model

Since we obtained data from multiple levels, we used Mplus7.4 (Muthén and Muthén, 2015) to test the proposed hypotheses. We first ran null models with no predictors, including only organizational identification as dependent variables. The ICC (1) value for the organizational identification as the dependent variable was 0.136, indicating that 13.6% of the variances in organizational identification reside between groups. The outcomes justified the inclusion of predictors in the group-level research.

### Hypothesis Test

Our theoretical model is multilevel, consisting of variables at both the team level (i.e., employee development-oriented organizational culture) and individual level (i.e., perceived overqualification, organizational identification, and creative performance). In testing the hypotheses, we conducted hierarchical linear modeling analyses because it can account for nesting in data while testing cross-level moderating effects. We tested cross-level moderating effects using slopes-as-outcomes models, controlling for employee education, age, gender, salary, and promotion chances at the individual level (i.e., Level 1). The cross-level analysis results are summarized at the bottom of Table 3. It can be seen from Table 3 that after concerning the control variables and main effects, the interaction influence of perceived overqualification and employee development-oriented organizational culture was significant with organizational identification (γ = 0.334, *p* < 0.01).

**TABLE 3:** Results for multi-level path analysis.

Outcome variable: OI	M1	M2	M3	M4
**Individual level**				
Intercept (γ_00_)	4.423**	4.405**	4.304**	3.880**
Age (γ_20_)		0.002	0.000	0.008
Gender (γ_30_)		0.025	0.044	0.073
Education (γ_40_)		−0.058	−0.024	0.019
Salary (γ_50_)		0.027	0.022	0.024
Promotion chances (γ_60_)		0.034	0.028	0.032
POQ (γ_10_)		−0.151*	−0.145*	−0.129*
**Team level**				
EDOOC (γ_01_)			0.316**	0.290**
POQ × EDOOC (γ11)				0.334**
σ^2^	0.169	0.146	0.145	0.145
τ_00_	0.014	0.014	0.004	0.004
τ_11_		0.046	0.009	0.003
Δ*R*^2^ within		13.61%		
Δ*R*^2^ between			71.43%	
Δ*R*^2^ between (moderate effect)				66.67%

*p < 0.05, **p < 0.01. EDOOC, employee development-oriented organizational culture; OI, organizational identification; POQ, perceived overqualification.

**FIGURE 2:**
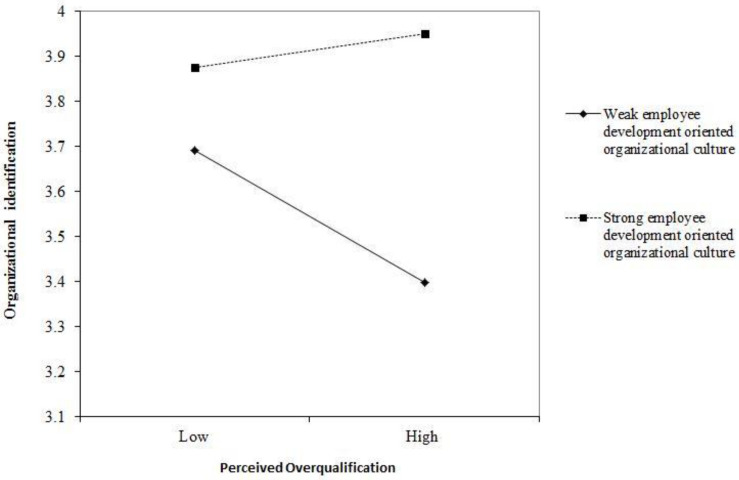
Employee development-oriented organizational culture moderates the relationship between perceived overqualification and organizational identification.

Figure 2 further shows this interaction by plotting the simple slopes when employee development-oriented organizational culture at 1 SD above and below its mean, such that perceived overqualification was positive to organizational identification while employees feel high employee development-oriented organizational culture (one standard deviation above), and it was opposite while employees feel low employee development-oriented organizational culture (one standard deviation below). Hence, hypothesis 1 was supported.

Hypothesis 2 predicts that organizational identification is positively related to creative performance. The results, as shown in Table 4, organizational identification was found to have a positive correlation with creative performance (γ = 0.364, *p* < 0.01). Therefore, hypothesis 2 was supported.

**TABLE 4:** The effect of organizational identification on creative performance.

Outcome variable: Creative performance	M1	M2
Education	−0.044	−0.057
Gender	−0.009	−0.056
Age	0.117	0.063
Salary	0.129	0.089
Promotion chances	0.026	0.012
Organizational identification		0.364**
*R*^2^	0.042	0.146**
Δ*R*^2^		0.104**
Δ*F*	1.451	19.692**

**p < 0.01. Two tailed test.

Hypothesis 3 predicts employee development-oriented organizational culture moderates the mediation effects of perceived overqualification on creative performance via organizational identification. The results based on repeated parameter sampling of Monte Carlo to estimate 95% confidence intervals showed in Table 5 that the indirect relationship in hypothesis 3 was supported. Specifically, perceived overqualification is positively and significantly related to creative performance when employee development-oriented organizational culture was strong [γ = 0.005, 95% confidence interval is (−0.023, 0.041)], but negatively related with creative performance when employee development-oriented organizational culture is weak [γ = −0.058, 95% confidence interval is (−0.130, −0.010), excluding 0]. Eventually, the difference between the two groups reached a significant level, and the 95% confidence interval (0.009, 0.151), excluding 0. Therefore, hypothesis 3 was supported.

**TABLE 5:** Monte Carlo parameter bootstrapping for conditional indirect effects.

	B	SE	95% CI	
			Low	High
High organizational culture (employee development) (+1 SD)	0.005	0.016	−0.023	0.041
Low organizational culture (employee development) (−1 SD)	−0.058	0.030	−0.130	−0.010
Difference between two groups	0.062	0.036	0.009	0.151

## Discussion

Drawing from the P-O fit theory, we predicted that perceived overqualification’s effects on creative performance through organizational identification and would be contingent on employee development-oriented organizational culture with both negative and positive effects. When employee development-oriented organizational culture is weak, the organizational identification of overqualified employees is low and leading to low creative performance. However, when employee development-oriented organizational culture is strong, organizational identification of those employees who feel overqualified is improved, and then drive the improvement of creative performance. We now discuss the theoretical and practical implications of the results.

### Theoretical Implications

Firstly, our study explains the mechanism of P-O fit and P-J fit promoting organizational identification by testing the interaction between perceived overqualification and employee development-oriented organizational culture on organizational identification. The results demonstrate that although perceived overqualification is a type of P-J misfit, its negative outcomes also can be reduced by P-O fit. By considering this issue, we emphasize the important impact of P-O fit on one’s work attitude and behavior. According to the P-O fit theory, we explain why overqualified employees may have contrary performance, and put forward the important role of employee development-oriented organizational culture in shaping organizational identification. Our result is consistent with the latest research statement of Erdogan (Erdogan et al., 2018a). Moreover, our study extends this prior work to demonstrate that organizational identification could be jointly determined by P-O fit and P-J fit.

Secondly, our study contributed to the perceived overqualification literature by identifying employee development-oriented organizational culture as an important boundary condition that influences organizational identification of overqualified employees. Employee development-oriented organizational culture indicates that situational factors can alleviate negative outcomes of overqualification. Previous findings on the relationship between perceived overqualification and performance were contradictory. Therefore, we believe that finding the boundary conditions of the relationship of perceived overqualification and its outcomes could determine whether it has a positive or negative impact. Although the previous study has pointed out that organizations can reduce the mismatch between overqualified employees and their jobs through job design (Wu et al., 2016). This study, from the perspective of organizational culture, found that building employee development-oriented organizational culture not only helps to improve creative performance but also reduces the cost of job design. Overqualification is a double-edged sword, this study provides a new research direction for the outcomes and contextual variables of perceived overqualification.

Thirdly, this study found that organizational identification is a key mediation mechanism between perceived overqualification and creative performance, which explained the contrary results of perceived overqualification. Overqualified employees produce positive/negative behaviors through their cognition, emotion, and attitude toward the organization. When overqualified employees have high levels of organizational identification, they will produce more creative performance, otherwise, they will have low creative performance.

Finally, the research on the influence of perceived overqualification on creative performance extended to the Chinese context. The overqualification phenomenon is serious in China. At the same time, the government encourages innovation. The research on the relationship between overqualification and creative performance is relatively lacking. This study expands the research of overqualification in China and provides a theoretical basis for organizations to reduce the negative impact of overqualification and respond to the call of promoting creative behaviors.

### Practical Implications

The present findings provide important implications for practice. Firstly, organizations should realize that although hiring overqualified employees may be associated with some negative effects but organizations can gain positive results by taking measures to enhance the organizational identification of overqualified employees. We found that employee development-oriented organizational culture could stimulate overqualified employee’s sense of organizational identification, then improve their creative performance. Specifically, Organizations should create an organizational culture that focuses on the growth and development of employees, such that, providing advanced training opportunities and assigning more challenging and meaningful work for the overqualified employees, and offering them with greater promotion space. In addition, the organizations should care for employees’ life to strengthen their affection attachment, undertake social responsibility and participate in public welfare to establish a good external reputation, which will all increase employees’ identification of the organization.

Secondly, to improve the creative performance of employees, organizations need to create an atmosphere that attaches importance to innovation and provide conditions conducive to innovation for employees. The organization can promote the construction of creative atmosphere and make employees perceive that the organization attaches importance to creative performance by making high incentive policies and increasing the investment of software and hardware about innovation. In addition, the organization should be tolerant enough to creative failure, so that overqualified employees have the opportunity to try and make mistakes without worrying about the punishment for failure.

Thirdly, we found that P-O fit could reduce the negative outcomes caused by P-J misfit. If the employee is too highly skilled for the job but shares the values of the organization, then some of the negative effects of perceived overqualification may be neutralized. The results suggest that P-O fit is an important moderator factor in the perceived overqualification-outcomes relationship, and organizations can benefit from considering these factors when estimating the potential impact of perceived overqualification. For example, when recruiting overqualified employees, organizations should focus on the matching degree between their values and the organization’s. If it is fit, organizations can make full use of the excessive qualifications after the introduction. If it is misfit, organizations should consider not to employ them. For the employees who have been employed, organizations could strengthen the learning and propaganda of organizational values for them, striving to achieve the P-O fit, and use P-O fit to offset the possible negative impact of P-J misfit.

### Limitation and Future Study

This study has a few limitations. Firstly, we explored only one boundary condition – employee development-oriented organizational culture to moderate the perceived overqualification-creative performance relationship. There may be other moderator variables to mitigate the negative impact of perceived overqualification, future research can explore other organizational factors to reduce the negative impact of overqualification. Secondly, although this study pointed out that P-J fit will interact with P-O fit to reduce the negative impact of perceived overqualification, which of them play a more significant role in this relationship is still a mystery. Therefore, where situational variables are affecting the relationship between P-J fit and P-O fit remains to be further studied. Thirdly, samples from Chinese enterprises limit the generalizability of the findings to different contexts. Cultural values can influence how individuals perceive and react to overqualification (Erdogan et al., 2011; Harari et al., 2017). As this study examined perceived overqualification in one specific cultural context, replicating the present investigation in other cultural contexts is worthy of consideration.

## Conclusion

Drawing on P-O fit theory, we explored the relationship between perceived overqualification and creative performance. The data were collected from 170 supervisor-subordinate dyads of 41 groups in 10 manufacturing companies across two time points in China. Results revealed that perceived overqualification is positively related to organizational identification when employee development-oriented organizational culture is strong but negatively related to organizational identification when employee development-oriented organizational culture is weak. Further, the indirect relationship between perceived overqualification and creative performance via organizational identification is moderated by employee development-oriented organizational culture. It widely accepted that as a type of P-J misfit, perceived overqualification may lead to negative consequences. However, the latest P-O fit theory shows that the fits of person is hierarchical and can affect each other (Lam et al., 2018), existing research begin to explore the most central fit type of all kinds of fit types (i.e., In our study, the negative outcomes of P-J misfit can be reversed by P-O fit). This finding will bring enlightenment to the management practices, future research can further explore the interaction of various fits such as person-job (P-J), person-group (P-G), and person-supervisor (P-S), person-environment (P-E), and so on.

## Data Availability Statement

The raw data supporting the conclusions of this article will be made available by the authors, without undue reservation.

## Ethics Statement

The studies involving human participants were reviewed and approved by the Institutional Review Board of Shanghai University, China. Written informed consent for participation was not required for this study in accordance with the National Legislation and the Institutional Requirements.

## Author Contributions

MZ, FW, and NL designed the study and revised the draft. MZ and FW collected the data. NL drafted the theory. MZ drafted the methods and results. All authors contributed to the article and approved the submitted version.

## Conflict of Interest

The authors declare that the research was conducted in the absence of any commercial or financial relationships that could be construed as a potential conflict of interest.

## Footnotes

1 https://www.wjx.cn
